# The Typability Index: A tool for measuring and controlling for typing difficulty in text stimuli

**DOI:** 10.3758/s13428-025-02877-y

**Published:** 2026-02-12

**Authors:** Emily A. Williams, Matthew Warburton, Martin Krzywinski, Faisal Mushtaq

**Affiliations:** 1 School of Psychology, University of Leeds, Leeds, UK; 2 Bradford Institute for Health Research, Bradford, UK; 3 Canada’s Michael Smith Genome Sciences Center, Vancouver, Canada; 4 Leeds Institute for Data Analytics, University of Leeds, Leeds, UK; 5 NIHR Leeds Biomedical Research Centre, Leeds, UK

**Keywords:** Typing, Keyboarding, Typing difficulty, Typability, Experimental control, Text stimuli, Shiny app

## Abstract

In typing proficiency tests, like those used in job recruitment or research studies, individuals are evaluated based on their speed and accuracy. However, the difficulty of the typed text, its ‘typability’, can impact typing performance, introducing variability that is unrelated to skill. To ensure valid comparisons across individuals, time, and conditions, it is crucial to control for this variation in text difficulty. To address this issue, we develop the Typability Index, a model that predicts the relative typing speed of text. Building on earlier attempts to quantify typing difficulty from the 1940s, we create a more advanced typability model using the 136 Million (136 M) Keystrokes Dataset (Dhakal et al., Proceedings of the 2018 CHI Conference on Human Factors in Computing Systems, 1–12, 2018), where over 168,000 participants each typed 15 sentences from a pool of 1,525 items. Through random forest regression, we identify eight key predictors from 30 candidate variables, including the proportion of lowercase letters, word frequency, and syllables per word. Trained on 80% of the dataset and validated on the remaining 20% and a novel dataset, the Typability Index explained 68–88% of the variance in typability, compared to the 34% explained by an earlier leading model (Bell, Unpublished Doctor’s Dissertation, University of Oklahoma, 1949). To promote higher control in typing research and assessments, we introduce a web-based tool to facilitate accurate measurement and fair comparisons of text typability.

## Introduction

### Why control for typing difficulty?

Imagine that you are taking a typing test for a job as a transcriptionist. You have consistently practiced your skill under anticipated test conditions and are confident that you can pass. However, during the test, you find yourself typing much more slowly than expected. While nerves could play a part, there could also be another reason: the text you have been given is unusually difficult to type. The degree of ‘typability’ of a given set of text can impact not only performance on typing tests but also outcomes of research studies that involve typing tasks.

The current research focuses specifically on copy typing tasks, where participants transcribe visually presented text rather than generating text themselves. Typing performance in copy tasks may differ from that in composition tasks, due to the reduced linguistic and semantic processing demands (Bonin et al., 2015, in handwriting). Nonetheless, even within copy tasks, the typability of the presented text remains a critical yet often overlooked factor.

Many experimental paradigms involve participants typing on a keyboard, often to compare performance across conditions. Some studies focus directly on the typing process, such as comparing typing training programmes (Donica et al., 2019), comparing typing on different input devices (Barrett & Krueger, 1994), or examining how factors like treadmill walking speed affect typing (Funk et al., 2012). Typing behaviour is also frequently used as a proxy to investigate unrelated factors, such as the effects of emotional induction (e.g., typing happy vs sad text; Maalej et al., 2022), or as a tool to assess recall of memorised words, for example in dual-task paradigms (Rossi, 2023).

In these experiments, varying text may be presented depending on the condition, either as an independent variable or to reduce practice effects from repeated text (e.g., Ruan et al., 2018). However, it is well established that certain features of the text itself can significantly impact typing behaviour (Salthouse, 1984, 1986). For example, the frequency of letter pairs (bigrams) in the language (e.g., ‘th’ vs ‘tv’; Behmer & Crump, 2017; Dvorak et al., 1936; Shaffer & Hardwick, 1969) and whether bigrams are likely to be typed with single or separate fingers or hands (Dhakal et al., 2018; Gentner, 1983) can influence typing speed. Therefore, comparisons between conditions may not be valid unless the relative ease of typing, or typability, is appropriately accounted for.

Failing to adequately control for typability has the potential to lead to at least three types of issues. First, researchers might find artificial differences between conditions if, for example, the ‘happy’ text set happens to be easier to type than the ‘sad’ text set. This could create a misleading impression that emotional content affects typing speed when the real cause was the text’s inherent typability. Second, not accounting for typability might mask true differences between conditions. For instance, if typing ‘happy’ text actually decreases typing speed but the text itself happens to be more typable, the true impact of emotional content could be obscured. Third, even when typing behaviour is not compared between conditions, inadvertently selecting text with extremely low (or high) typability can introduce floor and ceiling effects. Extremely easy or hard text may compress typing speed scores, making it difficult to accurately capture and compare typing abilities. These issues could undermine the validity and reliability of conclusions drawn from research involving typing.

#### How is typability currently controlled for?

Several text stimuli banks are available for typing-related research (e.g., Graff & Cieri, 2003; MacKenzie & Soukoreff, 2003; Vertanen & Kristensson, 2011), each positioned differently on the continuum between highly controlled and representative of real-world text. Some are specifically developed to investigate or compare text entry on various devices, a key focus in human–computer interaction. For example, MacKenzie and Soukoreff's (2003) phrase set includes text with minimal use of capitals and punctuation to standardise device comparisons, addressing variations in the steps required to insert these characters. Similarly, the InputLog multilingual typing test (Van Waes et al., 2019) requires no capitals or punctuation, though is a step forward in standardisation across languages. In contrast, other banks may contain naturally typed sentences that require adjustments for standardisation. For example, Dhakal et al. (2018) selected items from two such corpora (Graff & Cieri, 2003; Vertanen & Kristensson, 2011) and standardised sentence length as well as the number of capital letters and punctuation marks. While these adjustments aimed to manage text-related variables for consistency within their study, particularly in relation to international keyboard layouts, they were not specifically intended for comparing typing across different experimental conditions.

In studies that compare typing performance under different conditions, researchers may employ meticulous and labour-intensive strategies to address typability. For example, Pinet and Martin (2023) created two lists of 30 pseudowords and carefully matched them on features such as bigram frequency, hand/finger usage, and the number of letters. We employed a similar approach in upcoming publications, but while these methods yield precise control, the time and expertise required make them impractical for many research contexts. This complexity may explain why typability is not always adequately controlled for, highlighting the need for a Typability Index that consolidates the relevant text/key attributes that affect typing time into a single value.

#### Practical research applications of a Typability Index

A Typability Index would provide substantial benefit for research by offering enhanced control over the selection of text stimuli in research related to typing. It would enable researchers to fine-tune text selection for studies on typing behaviour or when typing serves as a proxy for other variables. For example, it could guide the selection of texts to ensure comparable typability across conditions, or differentiate texts by difficulty level. Additionally, typability scores could be integrated as a covariate in analyses where the text has already been selected or when other text features must be prioritised, similarly improving the signal to noise ratio around the true effects being studied.

Another key benefit of a Typability Index is the potential to reduce or remove the need for stringent, possibly artificial restrictions on text characteristics during stimuli selection, such as sentence length and the number of punctuation marks. Instead, researchers could compile a diverse text set varying in length, punctuation, capital letters, and other characteristics, and apply the Typability Index to match or contrast the typability between text set groups. This approach would enable the inclusion of more natural text in studies, rather than limiting choices to a predefined subset.

#### Previous attempts: ‘Typewritability’

In the 1940s and 1950s, there was interest in enhancing the reliability of typewriting assessments to accurately reflect changes in skill level. These efforts sought to quantify the so-called (at the time) typewritability and were primarily composed of unpublished theses and dissertations, with some attempts to construct a typing difficulty index based on one or more predictors (West, 1957). Bell (1949) constructed a Difficulty Index using 38 100-word excerpts typed by 89 typewriting students. The index, based on syllables per word, keystrokes per word, and percentages of frequently used words, was:

$$Difficulty\, Index=7.81 +3.49 \,syllables\, per\, word+ 0.08 \,percentage \,of\, frequently \,used\, words -2.44\, keystrokes\, per\, word$$

There are some stark differences between this typing difficulty index from the 1940 s and modern methods of controlling for typability. Factors like syllable count and word frequency, once integral to controlling for typability, are often overlooked in contemporary studies. Conversely, modern research tends to control for elements like bigram frequency and hand-finger usage, which were not yet considered in earlier indices. This contrast raises the concern that modern methods may sacrifice important predictors of typability for simplicity, potentially underestimating or ignoring key factors that affect typing performance.

#### Requirements for a successful Typability Index

For a modern Typability Index to be effective and enduring, there are arguably three important criteria it should meet.

##### 1. Consider a broad range of predictor variables

With advancements in research and computational power, we can now consider a broader range of predictors for typing difficulty than those identified by Bell (1949) and colleagues. The revival of typing research in the 1980s, driven by the advent of word processors and personal computers, explored various factors affecting typing at the inter-key interval level (i.e., the time between two key presses), as reviewed by Salthouse (1984, 1986). We will provide an overview of the known text/key attributes influencing typing speed across different eras of research and offer a thematic classification to ensure a broad range of predictor variables are considered when constructing the modern Typability Index.

##### 2. Utilise suitable datasets for model training, testing, and generalisation

It is preferable that high-quality datasets are used when training, testing, and assessing the generalisation of a Typability Index model. For example, training data should make use of a wide range of text items, each typed by a large number of participants at various typing speeds. Unfortunately, many existing typing datasets lack accessibility, text variety, or clarity in what text was actually presented. However, the 136 Million Keystrokes Dataset (Dhakal et al., 2018) provides a comprehensive resource that does not suffer from these shortcomings. This dataset includes data from over 168,000 participants who each typed 15 sentences from a pool of 1,525 items, ensuring text variety, robust sample sizes, and a range of skill levels per item. We will use this dataset to train and test the Typability Index. Additionally, we will validate generalisability with a separate and novel dataset. We previously collected data from around 100 volunteers, who each typed 15 movie quotes from the 1980s. This dataset will be described in more detail later and is openly available alongside this paper.

##### 3. Provide a user-friendly tool

Finally, for a modern Typability Index to be successful, it should be user-friendly and accessible. In the latter part of this paper, we will introduce the Typability Index web app. This Shiny app allows users to upload novel text and receive the predicted typability scores. These data are also available for over 1,000 sentences from the 136 Million Keystrokes Dataset (Dhakal et al., 2018), listing calculated rather than predicted typability scores. In addition, users can create text sets that are selected to have the same (or different) mean typability.

#### Research aim

In this paper, we introduce the Typability Index, designed to address the challenge of controlling for typing difficulty in research by predicting the relative typing speed, or typability, of input text. By employing advanced regression techniques and leveraging a broad range of candidate predictors and diverse datasets, we aim to offer a reliable and practical measure of typability. Our goal is to enable researchers to enhance their experimental control, either by ensuring the preferred typability levels across conditions or by incorporating typability as a covariate in statistical analyses.

Specifically, we develop the Typability Index model using random forest regression for feature selection and multiple linear regression for model building. We evaluate its predictive accuracy using both a subset of the Dhakal dataset and an entirely independent dataset, with the model explaining a substantial amount of variance in both cases. We also compare the Typability Index to Bell's (1949) Difficulty Index to demonstrate its improved predictive performance. Finally, we introduce the Typability Index web app, providing an accessible interface for researchers to apply the tool easily and effectively.

## Developing and testing the Typability Index

### Identifying the main text/key attributes influencing typability

In developing the Typability Index, we conducted a wide-ranging review of the text/key attributes that influence typing behaviour. This was based on prior empirical evidence of relevance to typing speed or effort, theoretical links to motor, cognitive, or linguistic processes involved in typing, and practical feasibility of measurement, i.e., ability to automate calculation. Table 1 presents a summary of the most influential attributes identified across the literature, providing rationale and supporting references. The table also includes potential influences that have not been, to our knowledge, previously investigated.

**Table 1:** Text/key attributes affecting typability, including classification and rationale

Text/Key Attribute	Description of metric	Rationale	Theme(s)
Attributes present in early typewritability indices (e.g., 1940s, 1950s)			
Keystrokes per word	Total keystrokes required, divided by total words*.	Shorter words are typically typed at a faster rate than longer words (Bell, 1949).	TP
Syllables per word	Total syllables, divided by total words*.	More syllables may slow text processing and typing (Bell, 1949).	TP
Word frequency	Percentage of top 1,000 English words, following Bell (1949).	High-frequency words may be processed and typed faster due to familiarity/practice (Bell, 1949).	TP, FoU
Total keystrokes	Total keystrokes required, including shift.	Longer text may be typed more slowly due to pausing to reread (West, 1957).	TP
Total words	Number of words in the text, identified as sequences of characters separated by spaces	Text with more words may be typed more slowly due to increased processing time (West, 1957).	TP
Punctuation marks	Count of punctuation marks, e.g.,.?!	Less frequent, typically further away, and may require additional keystrokes (e.g., shift; West, 1957).	FoU, L
Uppercase letters	Count of uppercase/capital letters.	Requires shift or caps lock, which is used less frequently (West, 1957).	FoU, L
Attributes since found to affect typing behaviour (e.g., 1980s)			
Hand categorisation	Number of bigrams (character pairs) that are: character repetitions; finger repetitions; hand repetitions; different hands.	Different categorisations are typically typed at different rates (Salthouse, 1984, 1986).	B, L
Bigram frequency	Average frequency of letter pairs in English.	High-frequency bigrams may be typed faster due to familiarity/practice (Salthouse, 1984, 1986).	FoU
Right-side keys	Proportion of characters’ keys on the right side of the keyboard.	Right hand is typically faster for most users (Dhakal et al., 2018).	L, B
Spaces	Count of spaces.	Spaces are generally typed faster, often by a thumb (Ostry, 1983).	L, FoU, B
Non-dictionary words	Number of words not recognised by standard dictionaries, including non-words, highly technical terms and typographical errors.	Likely typed more slowly due to unfamiliarity and text processing difficulty (Salthouse, 1984).	TP, FoU
Additional proposed attributes, not investigated previously to our knowledge			
Numbers	Count of numerical digits.	Less frequent and positioned away from the vertical centre.	L, FoU
Distance from home row	Average key distance from middle row of letter keys.	Greater distance may slow typing speed.	L

Themes are Text processing (TP), Frequency of use (FoU), Layout (L), and Biomechanical (B). *The number of words is customarily calculated as the number of characters, including spaces, divided by 5, which is used here unless stated otherwise. More specific derivations of the predictors considered for the model are presented in Appendix 1

While it is not feasible to include every text/key attribute ever explored in relation to typing speed, we have made a deliberate effort to cover a broad spectrum of influences on typability that capture motor, cognitive, and linguistic dimensions. To this end, we have categorised the influences into five interrelated themes that represent different aspects of typing behaviour, each of which is known or hypothesised to impact typability:

- **Text processing (TP):** How easily text can be understood, remembered, and re-read during typing; e.g., text with fewer syllables is generally typed faster than those with more syllables.
- **Frequency of use (FoU):** Relating to familiarity or practice, due to high occurrence levels; e.g., common letter pairs are typed faster than less frequent ones.
- **Layout (L):** Relating to the physical arrangement of keys on the keyboard; e.g., numbers, being further from the central area, are typically typed more slowly than other characters.
- **Biomechanical (B):** Relating to the physical mechanics of typing; e.g., letter pairs typically typed with the same finger, such as ‘ee’ or ‘de’, may be typed more slowly than those typically typed with different hands, such as ‘ei’.

### Method

#### Training and testing dataset

Here, we provide a summary of the pertinent features of Dhakal et al.’s (2018) 136 Million Keystrokes Dataset, but refer readers to the original paper for more details. As described in more detail later, the model was trained on 80% of this dataset and tested on the remaining 20%.

##### Participants

Dhakal et al.’s (2018) participants comprised 168,960 volunteers (52.7% female), with a mean age of 24.5 years (*SD* = 11.2), and 75% were between 11 and 30 years old (full age range not stated). Participants came from 218 countries, with 68% from the United States and 85% native English speakers. Participants’ mean typing speeds ranged between 4 and 158 words per minute (wpm), with a mean of 51.56 wpm (*SD* = 20.20 wpm).

##### Materials

Dhakal et al.’s (2018) set of 1,525 English sentences (of which we used 1,493, see Appendix 2) were sourced from the Enron Mobile Email corpus (Vertanen & Kristensson, 2011) and English Gigaword Newswire corpus (Graff & Cieri, 2003), with certain selection criteria applied by the authors. These criteria were a minimum of three words, a maximum of 70 characters, a maximum of four numbers, and only simple punctuation marks (,.!?’). These sentences included, for example, ‘1.5 million visitors will flood Atlanta each day of the Olympics.’, ‘Kim, here's the PSCO website address.’, and ‘What happened to the guy with the paper to sign?’. Regarding devices, 98% of participants typed on either a laptop-integrated keyboard or standalone keyboard, with the remainder using an on-screen/touch keyboard or small physical keyboard.

##### Procedure

Each participant typed 15 sentences, which were randomly selected from the bank of 1,525 sentences described above. Each presented sentence remained visible while typing, with produced text entered into a standard text field immediately below. Participants were instructed to read and remember the sentence, then type it as quickly and as accurately as possible. No restrictions were placed on the text field, meaning participants were able to use backspace and their typing was not constrained to only correct characters (i.e., they could continue entering text if they made a mistake). Participants pressed enter to submit their response.

##### Preprocessing

To arrive at the sample described above, Dhakal et al. (2018) excluded participants who had not completed all 15 sentences, their demographic information, and a questionnaire about their typing experience/strategy. Participants must have also achieved an error rate of less than 25%. Participants were excluded if there were likely distractions or technical problems, identified as any inter-key intervals (duration between consecutive keypresses) above 50 s. Of the 168,960 remaining participants, each of the 1,525 sentences (of which we will use 1,493; Appendix 2) were typed by 1,488–1,809 participants in the final dataset.

#### Generalisation dataset

To extend our out-of-sample testing, we assessed generalisability using a previously unpublished dataset collected by the lead author during unrelated pilot testing. This dataset was gathered through a gamified typing task that was promoted for the ESRC Festival of Social Science 2020 (UK), which provided participants with personalised statistics on their typing performance and insights into the benefits of efficient typing.

##### Participants

The group comprised 98 adult volunteers, with a mean age of 32.81 years (*SD* 10.43) and an age range of 19–62 years. Participant gender was not recorded. Participants’ mean typing speeds ranged between 16 and 102 wpm, with a mean of 64.26 wpm (*SD* = 17.03 wpm). A total of 330 participants began the task, but the current sample is limited to those who consented to have their data collected (which was not recorded otherwise) and those who completed the 15 sentences.

##### Materials

There were 15 sentences, which were quotations from popular movies from the 1980s. These sentences included, for example, ‘Where we’re going, we don’t need roads!’ (*Back to the Future*, 1985) and ‘Back off man, I’m a scientist.’ (*Ghostbusters*, 1984). The full set of sentences is presented in Supplementary Material B.

##### Procedure

Participants typed the 15 sentences in a random order. The presented sentence was displayed in the centre of the screen in the font OCR A Extended, wrapping to multiple lines as necessary. Prior to typing, the first character was displayed in white, with upcoming characters in pink. As participants typed a correct character, the typed character became blue, and the next character to be typed became white. If an incorrect character was entered (case-sensitive), there was no visual feedback, and the participant could not progress until the correct character was entered (no backspacing was required). Entering the final character correctly led to post-trial feedback in wpm, meaning pressing ‘Enter’ was not required to submit responses. Thus, the procedure differed slightly between the tasks used for the main test/train dataset and the generalisation dataset.

### Variables

#### Outcome variable: Typability

We first calculated the typing speeds for each participant's sentences in wpm. Following standard practice, the number of words was determined by dividing the total number of characters in the string, including spaces, by five (Wobbrock, 2007). The typing time was defined as the interval between the first key press and last key release of the sentence, including the final punctuation mark. Therefore, we divided the number of ‘words’ by the total time in seconds and multiplied this value by 60, yielding the gross wpm, which was not adjusted for errors.

Typability, or relative typing speed, was first calculated within each participant as the *z*-scored typing speed of each sentence they typed. Specifically, the *z*-score was computed as $z=\left(x- \mu \right)/\sigma$, where $x$ represents the typing speed for a given sentence, $\mu$ is the participant's mean typing speed across the 15 sentences they typed, and $\sigma$ is the standard deviation of their typing speed across these sentences. The *z*-score of the typing speed is a useful measure because it is independent of participant’s baseline typing speeds and is appropriate due to the relatively normal nature of the underlying distributions. The *z*-score indicates how much faster or slower the participant typed each sentence compared to their average, expressed in standard deviation units. Subsequently, the mean *z*-score for each sentence was calculated across the 1,488 to 1,809 participants who typed it.

#### Candidate predictor variables

The 14 text/key attributes identified in Table 1 were operationalised into a set of candidate predictor variables. For some attributes, multiple calculation methods were possible, resulting in more than one variable derived from a single attribute. For example, character type proportions could be calculated either as the proportion of total characters (e.g., proportion of characters that are lowercase) or as the proportion of non-space characters (e.g., proportion of non-space characters that are lowercase), to account for the distinct role that spaces play in typing (Salthouse, 1984). In other cases, a single candidate predictor variable was deemed sufficient for an attribute, such as syllables per word. This process produced a total of 30 candidate predictor variables, detailed in Appendix 1.

### Analysis

We removed 32 presented sentences that contained grammatical or typographical errors (see Appendix 2). To assess the potential influence of typing errors, sentence-level accuracy was calculated as 1 – (Levenshtein edit distance / presented sentence length), where the Levenshtein edit distance represents the number of insertions, deletions, or substitutions required to transform the typed sentence into the presented sentence (Levenshtein, 1966). The mean accuracy rate was then computed for each sentence. Accuracy was uniformly high: the sentence with the lowest mean accuracy was typed with 97.53% accuracy, and the median sentence had 99.08% accuracy. The mean accuracy across sentences was 99.04% (*SD* = 0.23%). As all sentences were typed with minimal deviation from the presented sentence, no sentences were excluded based on error rate. The remaining 1,493 sentences were randomly assigned to a training set (80%, *n* = 1,194) or a test set (20%, *n* = 299).

#### Model training

With the typability outcome variable and the candidate predictors calculated for the 1,194 training sentences, a three-stage feature selection process using the {randomForest} R package was undertaken to determine the final predictors for the model. Details of the random forest specifications can be found in Appendix 3.

##### Stage 1: Identifying the most explanatory calculation methods

A random forest regression was first conducted to determine which calculation method best captured each text/key attribute (e.g., proportion of lowercase characters vs. proportion of lowercase *non-space* characters). All 30 candidate predictor variables were entered, but attention was limited to those with more than one calculation method. Importance was determined by the increase in mean squared error (% Inc *MSE*) when a variable was excluded during the random forest process. The calculation method with the highest % Inc *MSE*, indicating the greatest impact on prediction accuracy, was selected as the preferred method. Other calculations for the same attribute were excluded from further stages.

##### Stage 2. Addressing multicollinearity and singularity

A second random forest regression was run with the 17 remaining candidate predictors. The % Inc *MSE* plot was used to determine the optimal number of predictors based on the inflection point, selecting 10 for the multiple regression model. Variables with a variance inflation factor (VIF) exceeding 10 indicated multicollinearity, while singularity occurred when candidate predictors had linear relationships (e.g., total keystrokes = number of characters / number of words), making it impossible to estimate unique coefficients. To resolve these issues, the least important candidate predictor(s) (in terms of % Inc *MSE*) in each problematic group was removed.

##### Stage 3. Selecting the final predictors

A third random forest regression was performed to determine the ideal number of predictors based on the % Inc *MSE* plot. From the remaining 15 predictors identified in Stage 2, nine were selected as optimal at the inflection point and entered into a multiple regression model. Multicollinearity was reassessed, and any non-significant predictors were excluded, leading to a final model with eight significant predictors. Given the large sample size, the *p*-value threshold was deemed appropriate to detect meaningful contributions to typability.

#### Model validation: Testing and generalisation

Actual typability scores and predictor variables were computed for the remaining 20% of sentences (*n* = 299) in the Dhakal dataset, representing the testing dataset. Predicted typability scores were then generated by applying the equation from the trained model to the sentences in this testing set, and these predictions were compared to the actual typability scores. The same procedure was followed for the generalisation dataset.

### Results

#### Typability scores

**Fig. 1:**
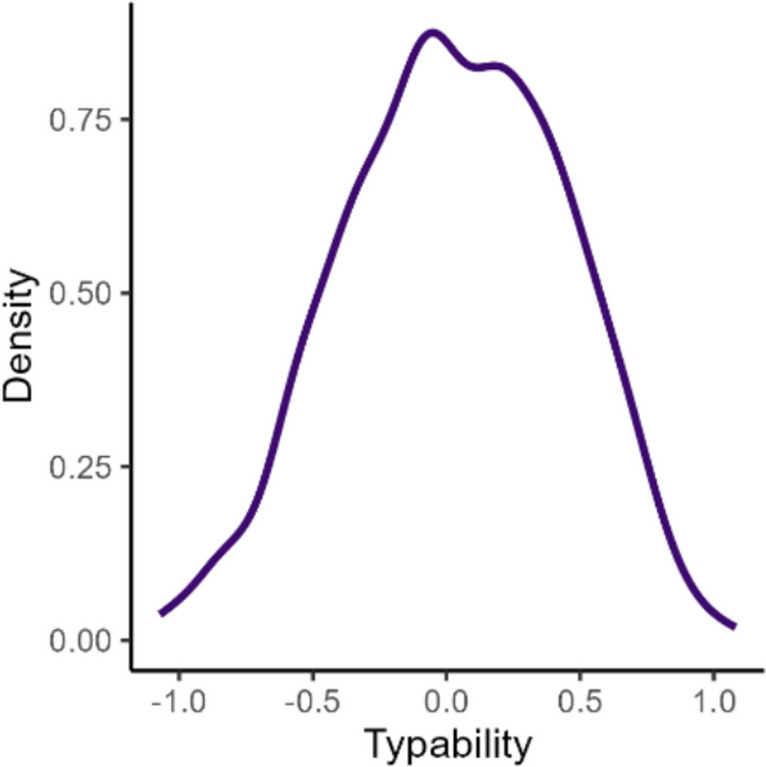
Distribution of typability scores in the training dataset

The distribution of typability scores in the training dataset is shown in Fig. 1. Table 2 provides illustrative examples of sentences for typability scores of 0, ± 0.5, and ± 1, along with a guide for typability score interpretations.

**Table 2:** Interpretations and example text for different typability scores

Typability	Interpretation	Example text
–1	1 *SD* slower than average	• The Senate should approve a 14-year-old treaty. • Suite 2750 in Downtown Denver.
–0.5	0.5 *SD* slower than average	• He started Sunday's game and had two catches for 70 yards. • I'll ask, he just came by.
0	Typed at one’s average speed	• I don't have the distraction of taking care of Mimi. • Do you want to fax it to my hotel?
0.5	0.5 *SD* faster than average	• Let me know if this is possible or where else I might find these. • The wind was strong and gusting.
1	1 *SD* faster than average	• I might have something at the office. • Thanks for sending this.

#### The Typability Index

**Fig. 2:**
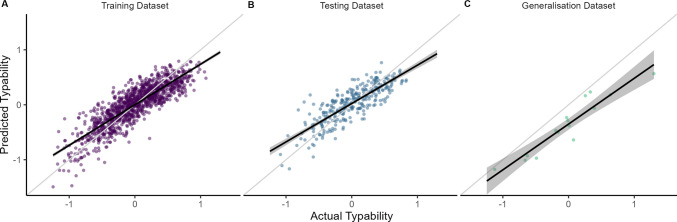
Predicted versus actual typability scores across datasets. The model was trained on the training dataset (**A**) and then evaluated on the testing (**B**) and generalisation (**C**) datasets to assess its predictive performance and generalisability. Light grey diagonal lines represent identity lines, indicating a perfect relationship between predicted and actual scores. Black lines denote linear regression lines between the predicted and actual scores, with dark grey bands representing 95% confidence intervals

Following the three-stage feature selection process using the training dataset, eight predictor variables were selected and entered into the multiple linear regression model. This model (*F*(8, 1,125) = 416.50, *p* <.001), with performance summarised in Fig. 2A and detailed in Table 3, accounted for approximately 74% of the variance in typability (adjusted *R*^2^ = 0.736), with prediction accuracy given by a root mean square error (*RMSE*) of 0.222.

**Table 3:** The eight predictor variables selected for the Typability Index

Predictor	Theme(s)	*β*	*B*	SE *B*	*t*	*p*
Proportion of lowercase non-space characters	L, FoU	0.533	4.694	0.207	22.68	<.001
Total keystrokes	TP	–0.433	–0.012	< 0.001	–22.34	<.001
Syllables per word	TP	–0.300	–0.431	0.028	–15.22	<.001
Proportion of words within high-frequency words	TP, FoU	0.266	0.693	0.052	13.35	<.001
Proportion of symbol non-space characters	L, FoU	–0.220	–4.037	0.433	–9.32	<.001
Bigram frequency	FoU	0.192	< 0.001	< 0.001	12.49	<.001
Proportion of characters within non-words	TP, FoU	–0.157	–1.665	0.163	–10.23	<.001
Proportion of right-side keys	L, B	0.100	0.462	0.076	6.08	<.001
(Intercept)			–4.022	0.193	–20.89	<.001

Themes, as described above Table 1, are Text processing (TP), Frequency of use (FoU), Layout (L), and Biomechanical (B). *β* represents the standardised beta coefficient, while *B* denotes the unstandardised beta coefficient and SE is standard error. Positive *β* and *B* values represent typing ease (faster than one’s own average) while negative values suggest difficulty (slower than average)

#### Validation: Testing and generalisation

The trained model was evaluated on the testing dataset, constituting the remaining 20% of the Dhakal et al. (2018) dataset. The model explained approximately 68% of the variance in this separate dataset (adjusted *R*^2^ = 0.682), maintaining the same predictive error rate (*RMSE* = 0.222). To assess external generalisability, the model was then applied to a novel generalisation dataset collected by the current authors, resulting in an adjusted *R*^2^ of 0.884 and *RMSE* of 0.399. This higher *RMSE* is due to a consistent underestimation of typability in this generalisation dataset. Figure 2 illustrates the relationship between the predicted and actual typability scores across the training, testing and generalisation datasets.

#### Comparison to Bell’s (1949) model

**Fig. 3:**
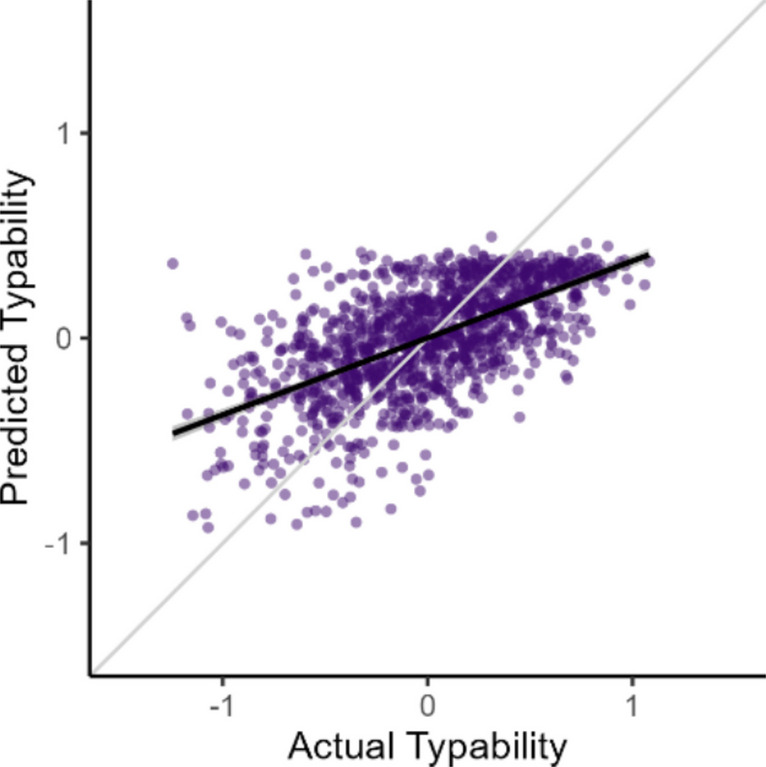
Predicted vs. actual typability scores for the Bell (1949) model, based on the training dataset. For additional details and context, refer to Fig. 2

The newly developed Typability Index was compared to Bell’s (1949) model, which, as described in the introduction, includes three predictors. To ensure a fair comparison, the predictor estimates from Bell’s model were refit to the present training dataset. The refitted Bell model (*F*(3, 1,190) = 238.10, *p* <.001), with performance summarised in Fig. 3 and detailed in Table 4, explained approximately 37% of the variance in typability (adjusted *R*^2^ = 0.374; *RMSE* = 0.343).

**Table 4:** A model of typing difficulty limited to Bell’s (1949) predictors

Predictor	*β*	*B*	SE *B*	*t*	*p*
Proportion of words within high-frequency words	0.479	1.249	0.075	16.55	<.001
Keystrokes per word	–0.236	–0.114	0.021	–5.53	<.001
Syllables per word	0.051	0.073	0.059	1.24	.215
(Intercept)		–0.462	0.123	–3.76	<.001

Although these two models are not nested, making an analysis of variance (ANOVA) comparison invalid, a comparison of Akaike information criterion (AIC) shows a clear advantage for the Typability Index. The Typability Index achieved a substantially lower AIC (–186.34) than the Bell model (839.92) and an intercept-only model (1,395.37), indicating a markedly better fit despite the increased model complexity. This supports the conclusion that the Typability Index provides a substantial improvement over prior approaches to estimating typing difficulty.

## The Typability Index web app

**Fig. 4:**
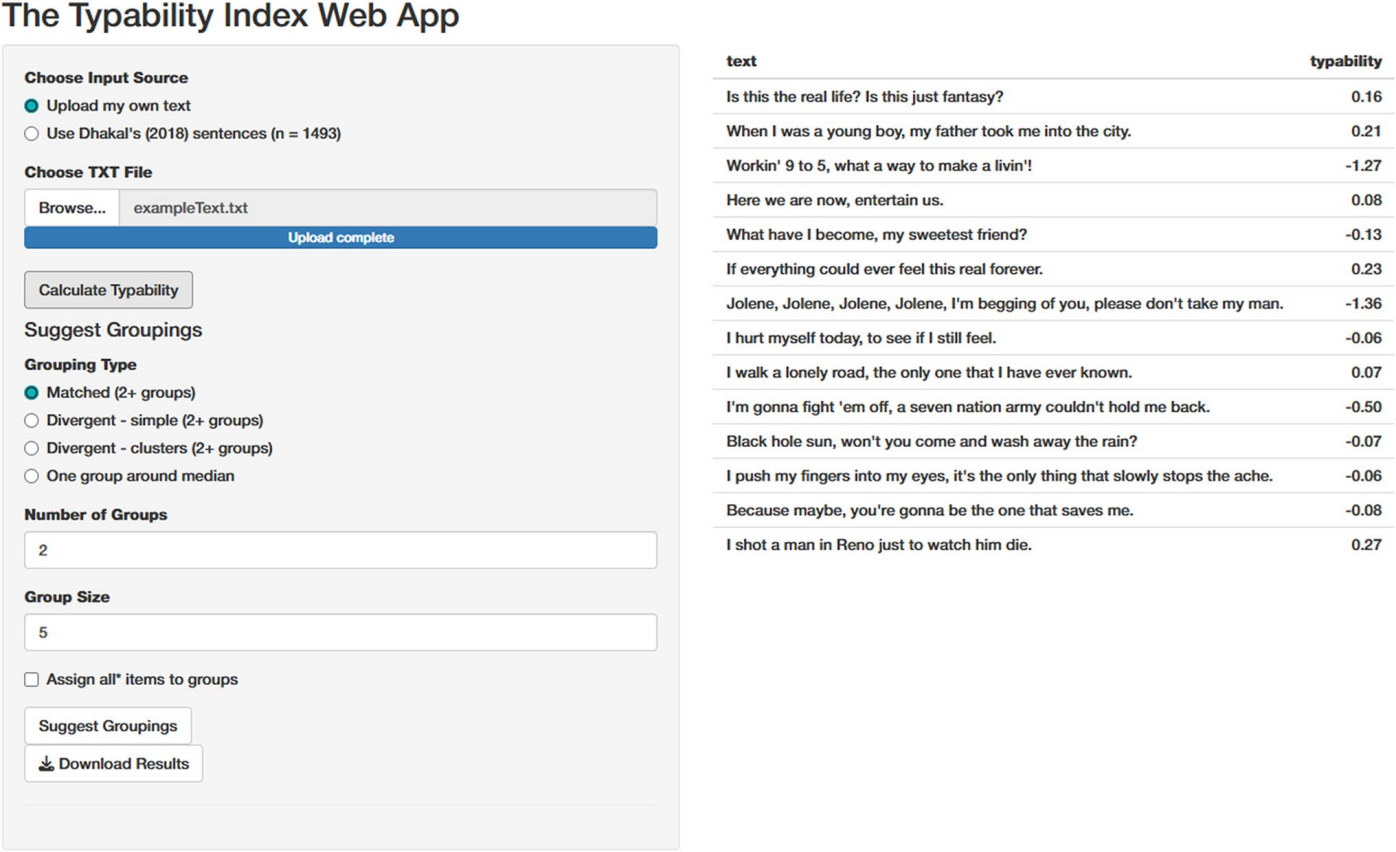
An example of novel sentences uploaded along with their predicted typability scores. The panel displays options for suggesting groupings based on grouping type, number of groups, and group size

The Typability Index is available as a user-friendly Shiny app (https://emily-a-williams.shinyapps.io/the-typability-index-web-app/), offering an interactive interface for calculating typability scores and generating suggested groupings of text stimuli. Users can upload novel text as a.txt file (as shown in Fig. 4) or access pre-calculated typability scores for the Dhakal et al. (2018) sentence set.

The app provides options for grouping text stimuli based on typability scores, allowing users to select from two types of groupings:

- **Matched groups**: This option aims to create groups with comparable mean typability scores. This is done by ordering items by typability and then assigning them sequentially to groups in a round-robin fashion. When group size is specified, this selection is centred around the median, where item density is typically highest (assuming a normal distribution).
- **Divergent groups – simple**: This option aims to create groups with distinct typability levels. For example, to form an ‘easy’ set and a ‘hard’ set, items are ordered by typability, then the specific number of items from the top and the bottom are assigned to different groups. For more than two sets, the middle groups are centred around the relevant quantiles, i.e., the 50th quantile (median) for three groups, and the 25th and 75th quantiles for four groups, etc.
- **Divergent groups – clusters**: This option also creates groups with distinct typability levels, but prioritises balancing the variance across groups. A specified number of clusters is first generated using K-means clustering, based on the desired number of groups. Items are then allocated to groups by selecting those closest to each cluster centre, helping to ensure that each group is both distinct in typability and reasonably consistent in spread across groups.

**Fig. 5:**
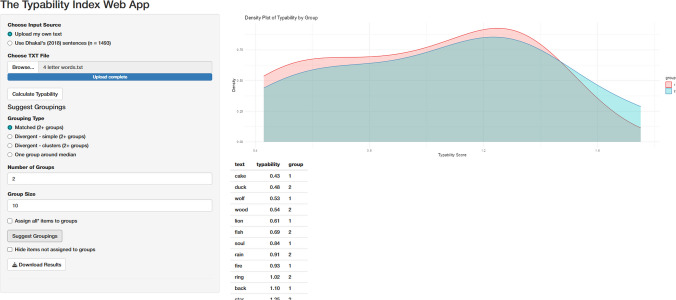
Suggested grouping for two groups of 10 four-letter words. Note that the user can scroll down to see the remaining group assignments, and download as a .csv file

These algorithms for group suggestions are designed to be simple for intuitiveness, but users are welcome to create custom groupings after exporting typability scores if they prefer. When using the suggested groupings, we encourage users to reflect on the underlying typability distributions of the text items and the output groupings, particularly with small samples of user-input text. The app provides a plot to help users visualise the distribution and composition of each group for this purpose. Figure 5 shows a use case of assigning 20 four-letter words to two different groups based on matched typability. In this case, users should input each word on a separate line in the input.txt file, without a header line. Finally, typability scores and optional group assignments can be downloaded as a .csv file.

## General discussion

Controlling for typability is essential in experimental research involving typing, as several text/key attributes are known to significantly influence typing behaviour (see Table 1). Many studies require participants to type under different conditions, but without adequately accounting for typability, comparisons between these conditions may not be fair. This could result in false positives, false negatives, and even when not comparing between conditions, floor and ceiling effects. The Typability Index was developed to simplify and enhance experimental control in typing-related research by accounting for the most important text/key attributes influencing typing difficulty within a single metric.

### Creation and validation of the Typability Index

We developed the Typability Index as a multiple regression model, which was trained on a randomly selected 80% of the sentences from the 136 Million Keystrokes Dataset (Dhakal et al., 2018). Our rigorous three-stage feature selection process identified eight key predictor variables (see Table 3), which collectively explain approximately 74% of the variance in typability with high prediction accuracy (*R*^2^ = 0.736, *RMSE* = 0.222). The top three most influential predictors were the proportion of non-space characters that were lowercase letters, total keystrokes, and syllables per word.

The Typability Index showed substantial improvement when compared to Bell’s (1949) model (*R*^2^ = 0.374, *RMSE* = 0.343), which contained three predictors and was fitted using the same training dataset. The AIC values further confirmed the superior fit of our model, demonstrating its enhanced predictive power and practical utility, even when penalising for additional predictors.

Validation of the Typability Index using the testing dataset maintained strong predictive power (*R*^2^ = 0.682, *RMSE* = 0.222). Furthermore, the model generalised well to a novel dataset, collected under different conditions by different authors, explaining approximately 88% of the variance (*R*^2^ = 0.884). While the predictive error increased in this separate and significantly smaller dataset (*RMSE* = 0.343), where typability was consistently underestimated, this reflected a shift in the intercept while the slope remained close to that of the unity line. This suggests a difference in the mean typability of the training and generalisation datasets (Fig. 2), perhaps due to the familiarity of the movie quotes in the latter. However, the relative typability of sentences within each set remained well predicted, which demonstrates consistent relative performance. Therefore, we recommend that researchers avoid mixing actual typability scores (of sentences in the training dataset) with predicted typability scores (from any novel text input). Instead, if researchers wish to select text stimuli on the basis of typability scores, they should depend only upon predicted or actual typability scores, not a mix of both. Overall, this validation shows that the Typability Index is robust and generalisable beyond the training data.

### Selected predictors of typability

Each of the eight predictors in the Typability Index contribute uniquely to typing difficulty. In order of importance, the predictors that increased typability (making typing easier) are the proportion of non-space characters that are lowercase letters, the proportion of words within high-frequency words, the average frequency of each bigram in the language, and the proportion of right-side keys. Conversely, the predictors that lowered typability (making typing more difficult) are the total number of keystrokes, the average syllables per word, the proportion of non-space characters that are symbols, and the proportion of characters that inhabit words without entries in English dictionaries (US, UK, CA, or AU). These predictors encompass a wide range of cognitive, linguistic, biomechanical, and motor processes, providing a comprehensive assessment of the text/key attributes that influence typing performance.

It is worth noting that some attributes that we expected to be predictive of typing difficulty were not selected for the final model during the feature selection process. The omission of the bigram finger/hand relations is particularly surprising, given the well-established differences in inter-key intervals for these bigram types (Dhakal et al., 2018; Gentner, 1983; Salthouse, 1984, 1986). This omission may be due to the fact that the relationship between the bigram types is not stable across skill level. That is, slower typists are typically faster at bigrams that constitute character repetitions than hand alternations, whereas this pattern is reversed in faster typists (Dhakal et al., 2018). Since the Typability Index was designed to be applicable across a wide range of typing speeds, with training data encompassing participants who typed between 4 and 158 wpm, other predictors may have been more descriptive, generalisable and relevant for representing the diverse typing population.

### The interactive Typability Index web app

In addition to developing the Typability Index, we developed an accessible tool to allow researchers to easily apply it to their research. We created a web app (https://emily-a-williams.shinyapps.io/the-typability-index-web-app/) that enables users to upload novel text or use Dhakal et al.’s (2018) sentences, calculate typability scores, visualise results, and download the typability scores and optional suggested groupings. Users can also create custom groupings after exporting typability scores if preferred.

### Practical applications of the Typability Index

The Typability Index and the associated web app provide several practical advantages for researchers of typing behaviour. The app enables more controlled sentence selection, allowing researchers to create text sets that (a) exhibit similar typability levels (matched groups) or (b) represent varying degrees of difficulty (divergent groups). In addition to these functionalities, typability scores can assist researchers in (c) avoiding floor or ceiling effects in typing performance, (d) potentially reducing the number of trials required for precise and reliable average typing speed measurements, and (e) alleviating traditional restrictions related to text length and punctuation, as overall typability can be effectively matched.

Typability scores can also serve as a valuable covariate in various research contexts. For example, in studies involving self-generated or 'free' text, it could help to distinguish between the cognitive and linguistic processes of text planning and the inherent difficulty of typing the text. In this case, users could record the typing time of the self-generated material, then compare it between conditions, using typability as a covariate. Additionally, in memory tests where participants type back lists of items, using typability as a covariate could mitigate potential confounds from differences in typing difficulty between lists, which might otherwise impact cognitive load and memory performance. This may ensure more accurate measures of recall.

Beyond research, the Typability Index has valuable applications for typing training and performance testing. For typing course developers, it allows for a progressive difficulty approach, presenting easier sentences initially and more challenging ones as learners advance. In the realm of typing tests for hiring or competitive typing, it ensures fairness by controlling for text difficulty, which is crucial for maintaining equitable test conditions and accurately assessing typing speed.

### Limitations

The Typability Index's generalisability is influenced by three main constraints inherent to the training dataset. Firstly, the model is based on Dhakal et al.'s (2018) dataset, comprising 1,525 English sentences sourced from the Enron Mobile Email corpus (Vertanen & Kristensson, 2011) and the English Gigaword Newswire corpus (Graff & Cieri, 2003), with certain selection criteria applied (e.g., no non-punctuation symbols). This somewhat limits the model’s applicability to other styles of text or other languages, as several predictors were calculated based on the (American) English language and keyboard layout. Secondly, the text unit of the dataset is sentences rather than paragraphs or single words. While this may affect the model's direct relevance to words or paragraphs, it is likely that the rank order of typability scores can still provide useful insights if calculated for a set of words or a set of paragraphs. This assumption is based on the fact that many linguistic and motor influences of typability at the sentence level also apply to smaller or larger text units. Finally, the dataset predominantly reflects typing on laptops and standalone keyboards, potentially limiting the model’s applicability to mobile devices.

A further limitation relates to the intended scope of the Typability Index. As noted, the tool is designed to calculate typability across a wide range of typing speeds, from 4 to 158 wpm, based on the training dataset. Consequently, it has not been tailored for any specific typing skill level.

Despite these limitations, the Typability Index provides a robust framework for controlling typing difficulty with confidence, aligning with its intended applications and the available data.

### Future work

Future developments could involve expanding the Typability Index to other languages, text lengths, and devices, where available training data allows. This would improve the Index’s applicability and accuracy across diverse linguistic and device contexts, further enhancing its utility in academic research and beyond. Additionally, researchers could explore the application of the current model in new settings, such as word list memorisation tasks where participants type back recalled items, or in studies involving self-generated text, to distinguish the cognitive processes of text generation from the typing difficulty influences covered by the model.

## Conclusion

The present work provides a practical solution to the enduring challenge of controlling text difficulty in research involving typing. The Typability Index enables researchers to select text stimuli based on specific typability criteria or account for typing difficulty by incorporating typability scores as a covariate. This advancement enhances experimental control, reducing the risk that variations in typing performance are confounded by text difficulty. By offering a refined method for managing text difficulty, the Typability Index can help deliver more meaningful and accurate evaluations of typing behaviour in research and beyond.

## Supplementary Material

The supplementary materials are available at https://github.com/EA-Williams/The-Typability-Index/ within the folder ‘SUPPLEMENTARY-MATERIALS’. The Typability Index Web App is available at https://emily-a-williams.shinyapps.io/the-typability-index-web-app/. We ask users to raise any questions or issues via GitHub Issues: https://github.com/EA-Williams/The-Typability-Index-Web-App/issues.

## Data Availability

The 136 M Keystrokes Dataset (Dhakal et al., 2018), which was used for model training and testing, is already publicly available at https://userinterfaces.aalto.fi/136Mkeystrokes/. The novel dataset used for testing generalisation is available at https://github.com/EA-Williams/The-Typability-Index/, along with minor amendments to a small proportion of datafiles the 136 M Keystrokes Dataset (See Appendix 2 for details), of which the original authors retain the rights. This repository also contains the code for creating and validating the Typability Index. This analysis was not pre-registered. The R project containing the materials and code for the Typability Index Web App is available at https://github.com/EA-Williams/The-Typability-Index-Web-App/. We ask users to raise any questions or issues via GitHub Issues. This R project is not required to use the Web App itself (https://emily-a-williams.shinyapps.io/the-typability-index-web-app/).

## Appendices

### Appendix 1: Calculations of the 30 candidate predictor variables

**Table:**

#	Candidate predictor variable	Calculation
1	Total characters	Total number of characters including spaces.
2	Total keystrokes	The minimum number of keystrokes needed to type the text accurately, assuming shift is used rather than caps lock.
3	Total words	The number of words in the text, defined as groups of characters separated by spaces, rather than the typical definition of words as five characters (when calculating speed).
4	Keystrokes per word	Total keystrokes divided by total words.
5	Characters per word	Total characters divided by total words.
6	Mean word proportion	1 divided by characters per word.
7	Proportion of words within high-frequency words	Number of words from the text that appear in the top 1000 words list* divided by total words.
8	Proportion of characters within high-frequency words	Number of characters that are contained in words from the text that appear in the top 1000 words list* divided by total characters.
9	Mean word frequency	Sum of the language frequencies of each word in the text, divided by number of words. Frequencies from SubtLEX_US_ (Brysbaert & New, 2009, ‘FREQcount’ variable).
10	Proportion of non-words	Number of words in the text that are not recognised in UK, US, AU or CA Hunspell English dictionaries (according to the {hunspell} package; Ooms, 2022) divided by total words.
11	Proportion of characters within non-words	Number of characters that are contained in words that are not recognised in UK, US, AU or CA dictionaries divided by total characters.
12	Syllables per word	Total number of syllables (according to the {quanteda.textstats} package; Benoit et al., 2018), divided by total words. This package uses the CMU Pronunciation Dictionary (Carnegie Mellon University, n.d.), and counts vowel clusters for words not in this dictionary.
13	Bigram frequency	Sum of the language frequencies of each letter pair in the text, divided by number of letter pairs. Frequencies based on Behmer and Crump (2017; 'Frequency' variable). This includes letter pairs only, with no spaces, and is based on approximately 3000 English language eBooks from Project Gutenberg.
14	Proportion of high frequency bigrams	Number of letter pairs from the text that are appear in the top 15 bigrams, divided by number of letter pairs. (An alternative approach akin to proportion of high frequency words). Frequencies from Behmer and Crump (2017).
15	Proportion of character repetitions	Number of character pairs relating to character repetitions (e.g. ‘rr’, ‘..’), divided by number of character pairs.
16	Proportion of finger repetitions	Number of character pairs relating to finger repetitions (e.g. ‘ed’, ‘k,’), assuming standard touch typing, divided by number of character pairs.
17	Proportion of hand repetitions	Number of character pairs relating to hand repetitions (e.g. ‘se’, ‘hi’), assuming standard touch typing, divided by number of character pairs.
18	Proportion of hand alternations	Number of character pairs relating to character repetitions (e.g. ‘qu’, ‘ty’), assuming standard touch typing, divided by number of character pairs.
19	Proportion of lowercase letter characters	Number of lowercase letters divided by total characters.
20	Proportion of uppercase letter characters	Number of uppercase letters divided by total characters.
21	Proportion of numbers	Number of numbers divided by total characters.
22	Proportion of symbols	Number of symbols (including both punctuation and non-punctuation symbols) divided by total characters.
23	Proportion of spaces	Number of spaces divided by total characters.
24	Proportion of lowercase letter non-space characters	Number of lowercase letters divided by total non-space characters.
25	Proportion of uppercase letter non-space characters	Number of uppercase letters divided by total non-space characters.
26	Proportion of number non-space characters	Number of numbers divided by total non-space characters.
27	Proportion of symbol non-space characters	Number of symbols divided by total non-space characters.
28	Keystrokes per character	Total keystrokes divided by total characters.
29	Proportion of right-side keys	Number of characters relating to keys on the right-hand side of the keyboard, assuming standard touch typing, divided by total characters.
30	Mean distance from home row	Sum of each character’s key distance from the eight finger resting keys on the home row, divided by total characters. Distances are based on Krzywinski (n.d.).

Predictors were calculated according to American English spellings and keyboard layout (ANSI) unless stated otherwise. *We used the 1,000 most frequent English words list from the Corpus of Contemporary American English (Davies, 2008-), including lemmatisations. For example, “do” is on the core list, so variations such as “doing,” “did,” and “done” were also considered. Including lemmatisations allows the Typability Index to capture familiarity with core concepts, not just the specific forms of words. This helps reflect both the cognitive familiarity and the ease of typing frequent or commonly recognised words

### Appendix 2: Present alteration of the 136 Million Keystrokes Dataset (Dhakal et al., 2018)

Upon close inspection, some presented sentences contained grammatical or typographical errors (e.g. “If your reasonable, I'll be reasonable.”; “That s all I have to say.”; “I think we are dong OK.”). This is understandable as part of the source was real emails (Enron). To remove the potential effect of these errors on typing behaviour, 32 sentences were excluded, identified by ‘Spelling and Grammar’ checks in Microsoft Word in addition to manual reading. See Supplementary Material A for the exact sentences removed and rationale for each.

In addition, minor manual alterations were made to 67 raw datafiles to fix parsing issues, mainly caused by participants typing Ctrl + M (shortcut for indent / new line) instead of Shift + M (see Supplementary Material A for details). Further, calculating participants’ mean typing speeds initially led to some negative values due to a relatively small number of two identified timestamp errors: (1) the recorded release time for some keystrokes preceded the recorded press time of the same keystroke; (2) for some trials, the first keystroke of the trial was apparently made before the end of the previous trial. To deal with these issues, any trials affected by the first timestamp error were excluded from the calculation of participants’ mean typing speeds because this timing error occurred mid-trial, suggesting the timestamps may not be reliable. Any erroneously-recorded keystrokes affected by the second time stamp error were removed, but the rest of the keystrokes for that trial were retained for analysis. This is because this second type of timestamp error related to only the first ‘keystroke’, which visual inspection suggested did not relate to the typing of the presented sentence that trial. In total, these alterations to account for timestamp errors affected 10,097 (<6%) of participant files. The adjusted datafiles accounting for parsing and timestamp errors are available on our Github repo (https://github.com/EA-Williams/The-Typability-Index/), with rights retained by Dhakal et al. (2018).

### Appendix 3: Specification of Random Forest Regression

We used the {randomForest} package, version 4.7-1.1, to run the random forest regressions. Deviations from the default settings included running 10,000 trees (ntree) instead of the default 500. Additionally, the number of variables randomly sampled as candidates at each split (mtry) was increased to 75% of the predictor count (i.e., 23.25) rather than the default 33% (10.33). Default values, including sampling with replacement (replace = TRUE), are detailed at https://cran.r-project.org/package=randomForest. Our full analysis code is available at https://github.com/EA-Williams/The-Typability-Index/.

## Declarations

### Generative AI

ChatGPT-4o (OpenAI, https://chat.openai.com/) was employed to improve the clarity and readability of sections of text. All AI-generated suggestions were critically evaluated and revised as needed to ensure text improvements remained scientifically accurate and consistent with the intended meaning.
